# Clinical trial reform in the post-COVID era

**DOI:** 10.1177/17588359231183676

**Published:** 2023-07-08

**Authors:** Meghan E. Mahoney, Srikala S. Sridhar

**Affiliations:** Department of Medical Oncology, Princess Margaret Cancer Centre, University of Toronto, Toronto, ON, Canada; Division of Medical Oncology, Princess Margaret Cancer Center, University of Toronto, 7-625 OPG, 610 University Avenue, Toronto, ON, Canada, M5G 2M9

**Keywords:** clinical trial, decentralize, oncology, real-world patient data, virtual

## Abstract

The COVID-19 pandemic precipitated the acute and efficient rollout of telehealth and virtual health care around the world. This review article focuses on the adoption of virtual care in the management of oncology patients, and discusses how virtual care offers the potential for large-scale, positive impacts on access to clinical trials. Virtual care during and following the peak of the pandemic has been found to be both safe and efficacious for oncology patients. Features, such as wearable health technologies, remote monitoring, home visits, and investigations being done closer to home, represent just some of the strengths of the virtual assessment rollout that were successfully utilized. One of the primary criticisms of oncological clinical trials is that clinical trial participants are not always representative of the patient populations treated in routine practice. This is in part due to stringent inclusion criteria and more broadly pertains to a lack of access to clinical trials, many of which are geographic as most trials are conducted in an urban, academic, or ‘centralized’ center. This paper seeks to discuss the barriers to clinical trial participation and to propose that the virtual care transformation that occurred during the pandemic has equipped oncological clinicians and researchers with the tools to better address these obstacles. A review of the literature on the impact of the virtual care rollout during and after the peak of the COVID-19 pandemic both locally and abroad was conducted. It is proposed that improving patient access through the decentralization of clinical trials has the potential to enhance evidence-based, real-world data, and to produce generalizable trial results that ultimately improve patient outcomes.

## Introduction

The nature and implementation of clinical trials has perhaps never been so crucial for the field of medical oncology. In the current and rapidly progressive era of targeted agents, immunotherapies, and adaptive cell therapies, it is essential that the principles of clinical trials be revisited and redefined to accommodate such evolution.^1^ Oncology patients have demonstrated improved overall survival during this time secondary to mass efforts and therapeutic advances not only in the pharmacological realm, but also from improved screening processes, surveillance, and advancements in surgical and radiation treatment techniques as well as enhanced supportive care.^2^ In addition to this, cancer care has become more individualized than ever before,^3^ and the ability to accrue and analyze mass amounts of data within and across trials has been revolutionized in the technological era. More recently, the COVID-19 pandemic prompted a wave of development and implementation of oncological patient care *via* virtual platforms within Canada and beyond.^4^ With this, it may be said that the pandemic serendipitously addressed a preexisting issue in medical oncology. That is, it provided clinicians and researchers with the tools to provide patients with access to what are known as ‘decentralized’ clinical trials. These are trials conducted primarily through virtual or telemedicine which are facilitated by local or mobile healthcare providers, allowing patients to participate in clinical trials beyond their primary cancer centers.^5^ The intent of this review article is to consider an in-depth assessment of the current landscape of clinical trials, particularly in the context of a pandemic-accelerated virtual world, to determine how access, eligibility, and real-world outcomes may be optimized utilizing these tools so that new standards may be established moving forward.

## Methods

The aim of this project was to conduct a review of the impact and outcomes of virtual care during the pandemic in the context of clinical trial participation, and to discuss how telemedicine can be utilized to improve access to clinical trials and the quality of data produced. Articles and guidelines to be included were searched *via* the PubMed database accessed through Memorial University Health Science Libraries. Key search terms included ‘virtual’, ‘oncology’, ‘clinical trials’, as well as ‘real-world patient data’, and ‘decentralized’ from 1990 to 2022 and articles of interest were selected. Full-text articles from reputable journals were reviewed and selected for this paper based on relevance. Frameworks, guidelines, and position statements were evaluated in the same manner and accessed on the dates noted in the reference section. General websites were accessed from search engines using the same search terms. The documents derived and included in this review are noted in the reference section accordingly. The analysis of the selected references was conducted by the primary author as well as the corresponding author who collaborated to produce Table 1 which was developed using the synthesis of information accrued.

**Table 1.** Challenges impeding access and generalizability to oncological clinical trials and proposed solutions in the post-COVID era.

Challenge	Type of barrier	Proposed solution	Limitations
Consent forms in English only	Access and generalizability	Central translational services including outreach (virtual/telephone)	Technological infrastructure and personnel, approval of remote/virtual consent
Distance to trial location site	Access and generalizability	Increased clinical trial implementation at community sites, virtual visits from clinician scientists at primary sites	Healthcare professional oversight at peripheral sites, resources per site
Access to treatment	Access	Drug-to-door shipment to patient homes	Pharmaceutical regulatory oversite, type of drug (oral)
Remote data collection (subjective)	Generalizability	Patient/user friendly remote technology (i.e. apps for smart phones, tablets) with education	Minimizing user error (i.e. language and age). Cost of remote tech
Remote patient monitoring (objective)	Access and generalizability	Home technology (i.e. home EKG readings)	Remote interpretation (i.e. cardiologist to read EKG), data accuracy
Stringent clinical trial criteria	Generalizability	Expand criteria to include minorities, the elderly, ECOG greater than 1	Patient safety, equitable access to digital health tech
Misinformation and mistrust	Personal	Improve transparency, patient and public engagement with the utilization of reliable social media sources. Patient champions/representatives	Resources and personnel to manage social media, public relations

ECOG, Eastern Cooperative Oncology Group; EKG, Electrocardiogram.

## The importance of real-world patient data and inequities in clinical trials

As it stands, phase I clinical trials are conducted to determine whether an investigational new drug that demonstrated efficacy in laboratory or cellular studies (sometimes described as phase 0 studies) may be safely applied to human participants. This explores the maximal tolerated dose a drug can be given with a reasonable safety and side effect profile, with the primary end point being dose-limiting toxicity. The established maximally tolerated dose from the phase I trial may then be carried forward to the phase II setting where a treatment will be evaluated for efficacy. Phase II trials will explore whether the investigational new drug of choice or treatment intervention demonstrates a statistically significant outcome (i.e. treatment response) at the indicated dose with reasonable adverse effects. If a promising result is demonstrated in the phase II setting, a phase III trial will then compare that intervention to the standard of care. A statistically significant result in a well-designed and robust phase III clinical trial has the potential (with replication and reproducible results) to reshape guidelines and treatment protocols for the indicated patient population.^6^ Phase IV trials, also known as post-marketing surveillance trials, may then be conducted. These studies often represent the first accrual of a much larger population of unselected oncology patients receiving treatment in routine clinical practice. Not all therapeutic interventions however are carried forward to a phase IV trial, and when they are, results do not necessarily represent the promising data initially cited in the phase III setting.^7^

This leads to the important concept of real-world patient data. As noted, oncological trials have been vastly criticized for their lack of generalizability which has become, if anything, more common in the context of therapeutic advances. That is to say that results demonstrated in phase III trials are not always replicated in the general patient population in routine practice, despite vigorous attention to validity, statistical power, randomization, and the fundamental features of a well-designed study. There are several reasons for this lack of external validity cited in the literature. First, to yield positive results, phase III trials tend to enroll patients who are able to tolerate treatment with its associated adverse effects, and thus are more likely to demonstrate a response. Eligibility criteria have therefore become increasingly stringent in this attempt to produce favorable outcomes and statistically significant results. While this enhances the internal validity of the study, it often excludes participants with comorbidities or a subpar performance status or measure of baseline fitness for treatment (i.e. Eastern Cooperative Oncology Group performance status higher than 1). This is detrimental to the generalizability of these studies as these unfavorable characteristics describe a significant proportion of the patient populations who will ultimately go on to receive these treatments once implemented into guidelines and clinical practice.

Moreover, it has been demonstrated in the literature that selection and population biases exist within clinical trials. Minorities and women, for example, are often underrepresented historically in clinical trial populations despite efforts to improve recruitment and representation among major organizations. The National Institutes of Health Act was established in the United States in 1993 to help improve minority representation and in 2009 it was modified such that all trials were required to report ethnicity.^8^ A study conducted by Duma *et al.* found that there had in fact been a reduction in the recruitment of minorities during a 14-year review period to follow, citing specific declines in the representation of African American/Blacks, Hispanics, and Indian American/Alaskan Native patients.^9^ There are noted to be logistical and practical factors contributing to this issue, such as language and financial barriers, but beyond this are complex sociocultural and religious factors that warrant significant attention.

Language barriers are a significant impediment to diversifying trial participants with several studies demonstrating that non-English-speaking patients are substantially less likely to enroll in clinical trials, even in cities with largely diverse populations.^10^ In part, this also speaks to the fact that most trial consent forms are written in English and non-English-speaking participants are excluded. It has also been demonstrated in the literature that patients who do participate in clinical trials exhibit favorable social determinants of health (i.e. higher socioeconomic status and levels of education), making this population more frequently represented than their socially disadvantaged counterparts.^11^ For example, a 2022 study on inequities and clinical trial enrollment in pancreatic cancer found that age, race, insurance, and geography were all barriers to enrollment. Even as enrollment increased during this study secondary to vigorous recruitment efforts, this increase in numbers was not demonstrated among African American patients and for patients on Medicaid.^12^

In addition, patient attitudes and beliefs have also been identified as barriers to clinical trial enrollment. A lack of trust in the medical system and academic institutions is particularly notable within minority populations and those who are socially disadvantaged. It is no coincidence that these populations also represent the medically underserved. These groups tend to carry an increased prevalence of malignancy making their participation in clinical trials crucial to providing optimal cancer care.^13^ It is not surprising that in the United States, Caucasian, middle class, married men are the most widely represented demographic in clinical trials.^14^ Among minority populations, particularly the African American population, there exists a generalized level of mistrust due to historical discrimination and mistreatment on an institutional level with one study noting that African American patients are more likely to express concerns about exploitation in the context of medical research.^15^ Without adequate primary care, the medically underserved are less likely to receive age-appropriate cancer screening leading them to present with more advanced disease. If an abnormality does get detected, these populations are often without the resources to attend the appropriate follow-up appointments required to obtain a timely diagnosis. In the United States in particular, these patient populations receive care from publicly funded institutions where they are less likely to have access to clinician researchers who could enroll them in clinical trials.^14^ It is clear that improving access is only one component of a complex issue pertaining to health disparity and underrepresentation of minorities in clinical trials. Building trust among socioeconomically disenfranchised and ethnically diverse populations will be an integral component to any improvement in recruitment initiatives, regardless of advancements in technology or infrastructure.

## Clinical trial access and enrollment

The need for more robust, real-world patient data calls for further assessment of the barriers that impede access to clinical trials among oncology patients. A 2018 cancer system performance report completed by the Canadian Partnership Against Cancer estimated that in Canada, participation of oncology patients in clinical trials ranged from 1% to 5.8% depending on the province. Newfoundland and Prince Edward Island were on the lower end of that spectrum and Alberta and Ontario on the highest. In the 10-year interval that was reviewed for this report, trial enrollment did not increase substantially within Canada between 2004 and 2014.^16^ The average trial enrollment was much lower than the enrollment reported in other advanced health care nations. In the United Kingdom, which is largely regarded as the exemplar of oncology trial enrollment, the National Health Service cites a participation rate of 14%. This came after the initiation of the National Cancer Research Network (NCRN) in 2000 which sought to foster access to cancer care and clinical trials *via* the preexisting designated centers within each region. The regional and institutional framework was established in the 1990s and consisted of a streamlined process to set up a structured referral system in each region facilitating access to specialized cancer care across the country. This expansive network seemed to be the ideal platform for the NCRN to conduct trials moving forward with substantial improvements cited in the years following, including an increase of 10.5% in the first 3 years after its initiation.^17^

The definition of access itself has evolved in the context of the COVID-19 pandemic which called for mass overhaul of and advancements in virtual care. This was implemented briskly out of necessity for ongoing patient care for this vulnerable population amid outbreaks and lockdowns. Canadian data that have explored this rapid transformation of care in the oncology world has demonstrated rapid uptake in addition to beneficial patient outcomes. For example, an assessment of the impacts of the COVID era on cancer care at the Princess Margaret Cancer Centre in Toronto demonstrated this rapid adoption of virtual assessments. It noted that just prior to the onset of the pandemic, less than 2% of clinic activities were conducted *via* a virtual platform. After enhanced technological capabilities were put in place in the month following declaration of the pandemic, widespread implementation of virtual care was established. In just 4 days after the rollout, the goal to convert 50% of in-person patient assessments to virtual was achieved, and this number increased to as high as 71% during the pandemic period. Pertinent components of cancer care (i.e. blood collection, home supports) were also performed remotely which helped to facilitate this transition.^18^

## Decentralized clinical trials

This transformation highlights the massive undertaking that was being achieved by cancer centers across Canada and around the world to minimize the risks of COVID and to ensure that patients were receiving continuous, optimal care during the COVID-19 pandemic. Accordingly, the next steps were to determine whether virtual care was associated with equivalent patient outcomes when compared to in-person clinic visits. Early post-pandemic literature suggests that replacing in-person assessments with virtual care was not detrimental to clinical efficacy or patient safety outcomes in cancer care.^19^ In fact, it has been suggested that oncology patients feel that virtual assessments provided them with care that is more appropriately tailored to their individual needs. A systematic review found that additional patient supports such as psychological counseling were as effective as in-person, and that genetic counseling provided *via* telephone was non-inferior to in-person care across a multitude of patients satisfaction measures.^20^ Moreover, remote access allows for more flexibility with scheduling and reduces accessibility concerns for many patients as it relates to their mobility, transportation costs, and psychological stress often associated with commuting to the cancer center – particularly for those living remotely.

It is worth noting that the term ‘access’ represents a multifaceted issue as it includes both patient barriers and institutional barriers that are reflective of the trial design itself. It is well established that the vast majority of clinical trials are completed in academic centers situated in urban settings. A 2017 global survey conducted by the Center for Information and Study on Clinical Research Participation found importantly that distance from the cancer center was one of two primary barriers for clinical trial enrollment – the other being lack of awareness.^21^ For this reason, recent studies have explored the need for decentralization of clinical trials. One 2022 study published in the *Journal of Digital Health* explains that traditionally, the logistics and implementation of clinical trials revolve around the center in which the trial is being conducted and describes this as the ‘site-centric’ approach. They propose that Decentralized Clinical Trials (DCTs) offer what can be instead termed a ‘patient-centric’ approach. This latter approach is more accommodating and would allow remote sampling and data collection from the comfort of the patients’ homes or other community-based hospitals or clinics that are much more convenient for patients and their caregivers. DCTs can include features such as virtual consent, local laboratory collections and imaging studies, and shipments of investigational drugs directly to patients as well as health technologies to support remote monitoring and home-nursing visits. This form of trial recognizes the need to widen eligibility criteria to encompass patient populations that are more reflective of our real-world populations. This includes patients with poor performance status, more comorbidities, lower socioeconomic status, mobility challenges, and those living further away from major cancer centers. This would likely result in trial results with more robust external validity. The aforementioned study collected survey responses from stakeholders including oncologists and oncology patients to assess their attitudes toward participation in DCTs. Overall, they found a significant willingness among all who were surveyed to move from site-centric to patient-centric care, understanding that this would require significant investment in health technology, resources, education, and training as well as appropriate navigation across legal and regulatory parameters.^22^

It should be noted that the necessity for decentralization of clinical trials was identified prior to the COVID-19 pandemic which accelerated the development of virtual care and its required infrastructure. A pre-pandemic, 2018 initiative by the FDA proposed a strategic framework to promote the use of real-world data that strongly supports the use of this type of healthcare technology. It highlighted ways to promote the development of patient-friendly, accessible trials intended to produce more generalizable results. Fundamental to this framework moving into 2019 was the plan to incorporate the use of real-world evidence to better inform regulatory decisions and practice guidelines. It stated that this would require significant advancements and investment in health technologies that would allow for remote patient access and monitoring, including virtual platforms that had the ability to connect with consumer devices.^23^ Conventionally, obtaining this type of post-marketing (or phase IV clinical trial) data would require the investment of millions of dollars and could take years to collect. The proposed framework surmised that leveraging data from cutting-edge health technologies would streamline this process to produce significant outcomes more quickly and efficiently while offering greater accuracy to real-world patient results. Despite this shift in framework, uptake in adopting DCT models was lagging. However, much of the technology to perform remote patient care and clinical trial implementation had been available for years though it was not adopted largely due to a lack of bureaucratic and systematic will. Little did anyone know that the true technological and operational push that was needed to implement this care would be borne of unfortunate circumstance in the years to follow. It may be said that the COVID-19 pandemic, despite all its detrimental consequences, has set the oncological research world up for the technological boost it greatly needed.

Given that virtual care has proven to be safe and achievable, the question remains as to whether it provides the field of oncology the real-world patient data that it requires. The European Society of Medical Oncology expert panel released a review article in 2022 supporting this exact notion. With patient safety and outcomes non-inferior to in-person visits, it is still essential to determine what this shift means for the patient experience. They commented on data obtained from the Melanoma Patient Network Europe. The responses were variable, with some patients reporting that they missed the in-person experience, particularly when it came to receiving bad news. Other patients, particularly given pandemic restrictions, were relieved to have this information delivered to them at home with family members at their side, which was not possible in many major centers during the pandemic. By and large, this expert review panel endorsed the adoption of DCTs and the patient-centered approach. It also speaks to some of the institutional and systematic macro-level considerations that will require significant oversight to propel this movement forward. Specifically, they recommend that this include the integration of systematic review boards across sites and countries that allow for expedited protocol amendments, faster drug approvals, and streamlining regulatory processes.^24^

## Discussion

This article intends to provide insights into the improvement of global standards of patient care in oncology, particularly in the realm of clinical trials. This review of the literature has shed light on several pertinent questions that oncology healthcare workers, administrators, and stakeholders must consider. A major criticism of phase III clinical trials is a lack of generalizability. The main reason for this is that real-world populations are either ineligible or not being included. This, combined with the issues of limited access and stringent eligibility criteria, further exacerbates the issue. Can the field of medical oncology utilize the virtual care transformation to ameliorate patient outcomes and address these issues simultaneously?

That is, if access to clinical trials may be improved through multicenter, virtual expansion outreach beyond the single urban center of expertise with revision and loosening of eligibility criteria, could it be possible to produce phase III results that are more easily replicated in phase IV/real-world patient settings? In addition, how can this be made possible within the literally and figuratively vast Canadian healthcare landscape?

With one of the most ethnically diverse populations in the world, increasing the variability and percentage of clinical trial patients in Canada while improving the validity of results could be revolutionary to cancer care within our country and beyond. There are relevant lessons to be gained from the rapid global response to the COVID-19 pandemic. A review of the National Insitutes of Health (NIH)-led research response to this critical era of medical research and development made building diversity in vaccine trials a leadership priority, knowing that the burden of the COVID-19 pandemic fell heavily on minorities. This review also calls for sustainable, large-scale global clinical trials networks. The pandemic highlighted the need for international coordination in addressing public health challenges and the same may be said for large-scale clinical trials.^25^ For example, global decentralization and remote patient access could allow for international clinical trials on rare cancer subtypes that would never have been able to accrue an adequate sample size in a single center to conduct a study with enough statistical power to produce meaningful results. While this would entail a significant overhaul of clinical trial access with virtual assistance particularly in community centers, it is quite possible that most of these institutions have undergone the virtual care reform secondary to pandemic restrictions. As was demonstrated after the initiation of the NCRN in the United Kingdom, if the infrastructure is already in place, then pouring time, resources, and efforts into substantially increasing enrollment in clinical trials is both practical and achievable.

**Figure 1.**
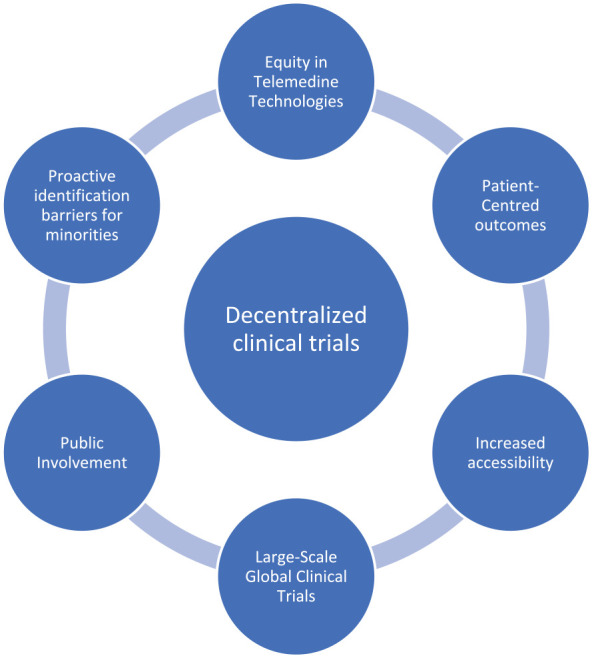
Pertinent considerations for optimal decentralized clinical trial design.

There have been some successful and noteworthy initiatives that focus on patient-centered research within the Canadian Cancer Trials Group (CCTG) which have been reported to improve clinical trial activity and enrollment within Canada in recent years. The work conducted by Needham *et al.* explains the inclusion of patients as meaningful partners in clinical trial design, with the patient at the center of the decision-making process. Proponents of this type of engagement seek to proactively identify potential patient barriers to participation during the recruitment period and address them. By inclusion of patient-centered end points (i.e. identifying outcomes that are important to patients in addition to quality of life), patients are motivated to enroll and continue in trials. With this focus on patient and public involvement, the CCTG has improved their trial enrollment and activity, noting the highest accrual and activity data in years.^26^ This highlights the importance of patient involvement and autonomy in their care, regardless of the platform. It is suggested that DCTs incorporate these patient-centered principles to facilitate inclusion. This is included in Table 1 which highlights several of the challenges discussed as well as possible solutions moving forward. Figure 1 reflects the major considerations which should be incorporated to design optimal decentralized trials.

It should be noted that this pursuit does not come without limitations, and it is prudent to discuss the perils of technological advancements in the information era. Interestingly, a 2022 study evaluated racial disparities in telemedicine among oncology patients during the pandemic and found that African American patients, uninsured patients, and those with lower socioeconomic status or living in rural areas were less likely to avail of oncological virtual care.^27^ This raises the concern that virtual care could worsen disparities if the factors propagating minority underrepresentation are not accounted for in DCT design. Trials should not operate under the assumption that all patients have equal access to telemedicine services (i.e. high-speed internet). It will be necessary that trials reliant on telemedicine and virtual data collection take these disparities into consideration and address them preemptively. In addition, it is imperative to consider the sociocultural beliefs and to address medical misinformation as the technological era has demonstrated its perils as was seen during the propagation of illegitimate medical information during the pandemic.

Although the utilization of virtual care has many benefits, there is also an element of depersonalization that comes with telehealth as reported by patients in post-pandemic surveys. When considering the nature of oncologist–patient relationships, difficult conversations (i.e. disclosing bad news regarding prognosis) require an amount of sensitivity that may be difficult to convey from behind a screen. Moreover, it is important to consider that the ability to properly use health-related technology might be lacking in some populations requiring the most inclusion (i.e. elderly, or non-English-speaking participants). Those patients who are less familiar with the use of mobile devices and applications could be limited in their participation if training and education is not adequate. Other limitations require the consideration of the burden that decentralization and enhancing access in the community may put on frontline healthcare workers who are already experiencing the brunt of strain of the Canadian healthcare system. Physicians and nurses serving remote and rural populations would play a fundamental role in facilitating remote care; however, this will not be possible without the infrastructure, resources, and supports required. This brings about the element of cost for these key components which go beyond technological investments and would also have to include highly specialized training of healthcare professionals to conduct remote care and data collection safely and effectively. There are notable risks to storing patient data virtually as well. In the age of healthcare system hacking and breaches of confidential patient information, ensuring the appropriate regulatory protocols and digital security system checks will be a crucial element of the patient-centered, virtual clinical trial. This may limit some of the obvious advantages of widespread, virtual coordination across different regions if regulatory bodies have restrictive access pertaining to the sharing of patient information.

While the systemic and technological means to move toward patient-centered clinical trials exist, to see meaningful increases in enrollment, efforts must also be concentrated on gaining patient trust. As noted, patient attitudes, specifically the marginalized and medically underserved, reflect a lack of trust in clinical trials and the medical system in general. It will be essential to create meaningful initiatives in this regard such as campaigns or endorsements for well-known individuals who could serve as advocates and champions for clinical trial participation. While this paper focuses on the potential advantages of telemedicine, it is essential to pay close attention to the potential perils of virtual trials to mitigate propagation of inequity in the setting of telemedicine which has the potential to counteract the intent of improving minority representation.

While the transition to decentralized, patient-centered clinical trials would undeniably be a significant undertaking within Canada, the rapid uptake of virtual care during the COVID-19 pandemic serves as both an example and a means to provide the level of care necessary to have this come to fruition. This would certainly not lessen the importance of the randomized control trial, but to synthesize it with real-world patient data and evidence that was often lagging in time and initiative. With the support of key stakeholders, practitioners, and health authorities, there is every reason to believe that with the amelioration of clinical trial access, enrollment and thereby results, that patient-centered trials have the potential to improve cancer care outcomes for the people who so greatly deserve this effort, our patients.

## Declarations

**Ethics approval and consent to participate:** Not applicable.

**Consent for publication:** Not applicable.

**Availability of data and materials:** Not applicable.
